# Determinants of Oral Health Outcomes and Quality of Life in Diabetic Patients from Western Romania: A Behavioral Model Approach

**DOI:** 10.3390/dj13060247

**Published:** 2025-05-30

**Authors:** Iulia Alexa, Ramona Dumitrescu, Doina Chioran, Octavia Balean, Vanessa Bolchis, Ruxandra Sava-Rosianu, Simona Popescu, Atena Galuscan, Daniela Jumanca

**Affiliations:** 1 Translational and Experimental Clinical Research Centre in Oral Health, Department of Preventive, Community Dentistry and Oral Health, “Victor Babeș” University of Medicine and Pharmacy, Eftimie Murgu Sq. No. 2, 300041 Timisoara, Romania; iulia.alexa@umft.ro (I.A.); dumitrescu.ramona@umft.ro (R.D.); vanessa.bolchis@umft.ro (V.B.); galuscan.atena@umft.ro (A.G.); jumanca.daniela@umft.ro (D.J.); 2 Department of Dentistry, Faculty of Dental Medicine, “Vasile Goldis” Western University of Arad, 310045 Arad, Romania; 3 Clinic of Preventive, Community Dentistry and Oral Health, Department I, “Victor Babeș” University of Medicine and Pharmacy, Eftimie Murgu Sq. No. 2, 300041 Timisoara, Romania; 4 Department of Anesthesiology and Oral Surgery, “Victor Babeș” University of Medicine and Pharmacy, Eftimie Murgu Sq. No. 2, 300041 Timisoara, Romania; chioran.doina@umft.ro; 5 Second Department of Internal Medicine, “Victor Babeș” University of Medicine and Pharmacy, Eftimie Murgu Sq. No. 2, 300041 Timisoara, Romania; popescu.simona@umft.ro; 6 Department of Diabetes, “Pius Brinzeu” Emergency Hospital, 300723 Timisoara, Romania

**Keywords:** oral-health-related quality of life (OHRQoL), diabetes mellitus, periodontal disease, behavioral determinants, Andersen’s behavioral model, health disparities, dental care access

## Abstract

**Background/Objectives**: Oral health and diabetes are closely linked through shared inflammatory, behavioral, and socioeconomic factors. This study examined the determinants of oral health outcomes and oral-health-related quality of life (OHRQoL) in Romanian diabetic patients using Andersen’s Behavioral Model. **Methods**: A cross-sectional study was conducted in early 2025 among 79 diabetic patients at a public clinic in Western Romania. Data were collected through questionnaires, clinical oral exams, and the OHIP-14 instrument. Variables were analyzed using Andersen’s Behavioral Model and standard statistical tests, including regression and correlation analyses. **Results**: Participants had a mean age of 61.2 years; 86.1% had type 2 diabetes and 13.9% type 1. Over 49% reported gingival bleeding, and 38% experienced dental sensitivity. Regression analysis identified limited awareness (OR = 2.21, *p* = 0.033) and low income (OR = 1.89, *p* = 0.041) as significant predictors of periodontal symptoms. OHIP-14 scores were weakly correlated with glycemic control (r = 0.17) and dental sensitivity (r = 0.16) but not with objective periodontal parameters. Rural residence, lower education levels, and poor awareness were associated with reduced service utilization and poorer perceived oral health. **Conclusions**: This study highlights the impact of behavioral, clinical, and socioeconomic factors on oral condition and OHRQoL. Oral health should be integrated into chronic disease care, with prevention-focused strategies aimed at improving access and reducing disparities, especially in older and rural populations.

## 1. Introduction

Oral health is a fundamental component of general health and quality of life, and yet it continues to be one of the most overlooked areas in public health, despite its well-established links to systemic conditions. The Global Burden of Disease Study (2019) estimates that oral diseases affect nearly 3.5 billion people worldwide, making them among the most widespread health problems. Untreated dental caries remains the most prevalent condition globally, while periodontitis, oral cancers, and edentulism have substantial impacts on individuals’ well-being and contribute significantly to social and economic health disparities [1]. The 2030 Agenda for Sustainable Development, adopted by the United Nations, emphasizes universal health coverage and the integration of non-communicable disease (NCD) prevention, including oral health. Complementing this, the World Health Organization’s 2030 Oral Health Global Strategy provides a dedicated framework for improving oral health equity and access worldwide [2].

Diabetes mellitus (DM) is not only a systemic metabolic disorder with wide-reaching consequences, but also a condition deeply intertwined with oral health. A growing body of research supports the bidirectional relationship between diabetes and periodontal disease, mediated through mechanisms such as chronic inflammation, impaired immune responses, and oxidative stress [3]. Poor glycemic control accelerates periodontal breakdown, while periodontal infection further compromises metabolic regulation. This interdependence has direct consequences for both systemic health and oral-health-related quality of life (OHRQoL).

OHRQoL has emerged as a vital component of comprehensive patient care. It reflects the functional, emotional, and social impact of oral health on individuals’ daily lives. For patients with diabetes, oral complications such as bleeding gums, tooth mobility, xerostomia, and delayed healing can negatively influence self-perception, social functioning, and mental well-being [4]. Yet, in Eastern European countries like Romania, research on OHRQoL among individuals with DM remains scarce, and public awareness about the oral–systemic connection is often limited [3,4].

Over the past few decades, numerous risk factors have been associated with the development and progression of periodontal disease (PD), including increasing age, male gender, lower socioeconomic status, limited educational attainment, diabetes mellitus, smoking, poor oral hygiene practices, and psychosocial stressors [5,6,7,8].

Among these, diabetes mellitus—both type 1 and type 2—is particularly relevant, given its well-established bidirectional relationship with periodontal inflammation.

This connection becomes even more critical in aging populations, where both conditions tend to coexist and mutually exacerbate one another. While extensive research has highlighted the negative impact of PD on OHRQoL [9,10,11,12], evidence also supports that periodontal therapy can lead to significant improvements in functional, psychological, and social aspects of oral well-being [13]. Moreover, lifestyle behaviors and awareness regarding periodontal disease are closely linked to oral hygiene habits and service-seeking behavior [10]. While T2DM is more prevalent in older populations and frequently associated with comorbidities, T1DM typically has an earlier onset and may lead to long-term cumulative effects on oral health. Both subtypes require targeted oral health strategies adapted to their specific clinical and psychosocial profiles.

In recent years, Andersen’s Behavioral Model (ABM) has been widely utilized to investigate the multifaceted factors influencing healthcare access and utilization. This model considers predisposing characteristics, enabling resources, and perceived need as key determinants of health service use. ABM has been effectively applied to understand patterns of dental service utilization and to explore how various factors impact oral-health-related quality of life (OHRQoL) [14,15], as well as its connections to chronic systemic diseases such as diabetes [16,17]. Originally developed to explain how individuals’ use of health services is shaped by a combination of social, behavioral, and attitudinal factors, ABM offers a robust structure for analyzing health outcomes within a multidimensional context. More recently, ABM has been applied in dental research to assess oral health behaviors and access to dental care, taking into account psychosocial variables, cost-related barriers, and perceived need [15,18,19]. These applications have emphasized the relevance of this model in identifying key determinants that influence not only service utilization, but also broader oral health outcomes. In this context, the use of multidimensional frameworks like Andersen’s Behavioral Model offers a valuable perspective for understanding how individual, behavioral, and structural factors contribute to disparities in oral condition and access to care.

In light of these advantages, Andersen’s Behavioral Model was selected as the conceptual framework for this study. Its established use in oral health research—particularly in exploring dental service access, disparities, and health outcomes—made it especially relevant for our investigation. Recent studies have successfully applied this model to oral healthcare behavior in vulnerable populations, including diabetic patients and underserved groups [20,21], further supporting its applicability in our context.

Recent European studies have further emphasized the role of broader socioeconomic transitions and psychosocial barriers in shaping oral health behaviors and access to dental care. For example, Baumeister et al. (2024) [22] demonstrated that retirement, educational background, and occupational history significantly influence both perceived oral health and the use of dental services among older adults across 31 countries, including Romania. Additionally, a systematic review by Pabbla et al. (2021) [23] identified oral health literacy deficits, financial hardship, and acculturation-related stress as major barriers to care among marginalized populations in Europe.

The delayed diagnosis of oral diseases is often attributed to limited awareness of their symptoms and insufficient financial resources [24]. Previous studies conducted in Western Romania have shown that oral hygiene practices in persons affected by diabetes are often suboptimal. Over half of patients report avoiding dental visits altogether, primarily due to financial barriers or lack of perceived need. Moreover, less than 40% brush their teeth twice daily, and the use of interdental aids is rare [4]. In this context, poor oral health behaviors compound the already elevated risk of periodontal disease among diabetic individuals, exacerbating both systemic complications and quality of life impairments.

Romania’s healthcare system presents substantial structural and financial barriers to equitable dental care access. Although the National Health Insurance House (NHIH, Bucharest, Romania) provides limited reimbursement for basic dental services, many essential procedures—including periodontal therapy, prosthetic rehabilitation, and endodontic treatment—remain uncovered. Approximately 99% of dental care is delivered through private practices, and while some of these clinics are authorized to offer reimbursed services through the NHIH, the allocated funding is minimal and tightly regulated. Only about 1% of services are delivered in public settings such as dental faculties or emergency dental offices within university hospitals. Moreover, the reimbursement ceiling for each dental provider varies by specialization and practice location (urban vs. rural), but it rarely exceeds the equivalent of EUR 900 per month. In practical terms, this amount is insufficient to cover the costs of more than one or two complex cases per month, such as those involving prosthodontic treatments [24]. Consequently, access to comprehensive dental care remains highly restricted, particularly for patients with chronic conditions requiring ongoing management, such as those with diabetes. These limitations are further exacerbated in rural areas, which are often underserved by dental professionals and where private dental care may be inaccessible due to cost and distance. With over 75% of practices concentrated in urban settings, oral healthcare in Romania risks becoming a privilege rather than a right. This disparity is especially relevant for elderly patients and retirees, and whose modest or fixed incomes often prevent them from pursuing either preventive or therapeutic dental interventions. For medically vulnerable populations, these intersecting barriers significantly impair the likelihood of receiving timely, adequate, and affordable oral healthcare.

Population aging represents a critical public health challenge worldwide. By 2030, one in six individuals globally is expected to be aged 60 years or older [25]. As life expectancy increases, the prevalence of chronic diseases such as DM also rises, placing significant strain on healthcare systems. In older adults, diabetes is frequently associated with multiple comorbidities and functional limitations, which can affect both general and oral health status. A growing body of literature has examined the relationship between aging, chronic illness, and healthcare utilization, with particular attention to self-rated health, access to services, and mortality outcomes [22,26,27]. However, many of these studies have not included oral health indicators in their assessment frameworks, and dental care is often excluded from broader healthcare utilization analyses. This oversight is particularly important given the strong bidirectional association between diabetes and periodontal disease, which has been well documented, yet remains under-addressed in public health planning.

The present study contributes to this evolving field by investigating oral health status and dental service utilization among Romanian diabetic patients—including both type 1 and type 2 diabetes—within a healthcare system characterized by regional disparities and limited public coverage of dental services. The research was carried out in Western Romania, a region marked by pronounced socioeconomic disparities. While Timișoara, the main urban center, benefits from advanced healthcare infrastructure and a high concentration of specialized chronic care services, many rural areas struggle with poverty, limited medical access, and reduced health literacy. These systemic contrasts in education, income, and healthcare coverage created a suitable context for exploring the multifactorial influences on dental care utilization among patients with DM. Romania’s national health insurance system provides partial coverage for dental services, including basic preventive procedures such as dental cleanings (scaling and polishing) and extractions. However, more complex or long-term treatments—such as advanced periodontal therapy, restorative interventions, or prosthodontics—are not reimbursed and must be paid out-of-pocket. Given that over 75% of dental practices operate in the private sector, the cost of comprehensive dental care remains a substantial barrier, particularly for elderly patients relying on fixed retirement incomes. Furthermore, there is a marked urban–rural disparity in the distribution of dental clinics. According to recent national health reports, urban areas host up to six times more private dental offices than rural areas, contributing to unequal access to oral healthcare across regions. These combined financial and geographic barriers contribute to the underutilization of dental services among diabetic patients living in underserved areas of Western Romania.

Given the increasing clinical recognition that both type 1 and type 2 diabetes mellitus contribute to oral health deterioration through chronic inflammation, impaired immune response, and systemic metabolic dysregulation, this study included adult patients with either form of diabetes. Although these conditions differ in pathophysiology and onset age, they share common oral health complications and structural barriers to care, making their joint inclusion relevant for understanding access disparities and behavioral determinants in diabetic populations.

In this context, the present study investigates the oral health status and dental service utilization among Romanian patients diagnosed with both type 1 and type 2 diabetes mellitus, taking into account the structural limitations of the healthcare system, barriers to care access, and individual behavioral differences. By applying Andersen’s Behavioral Model as the conceptual framework, this research aims to identify key determinants—individual, clinical, behavioral, and structural—that influence both oral health outcomes and oral-health-related quality of life (OHRQoL).

The objective of this study is to provide an integrated and context-sensitive understanding of how the interaction between diabetes type, personal characteristics, and systemic barriers shapes oral condition and perceived quality of life in a medically vulnerable population. This approach aims to inform targeted clinical and educational strategies tailored to the realities of oral healthcare in Romania.

## 2. Materials and Methods

### 2.1. Ethics and Study Design

This observational cross-sectional study was conducted between February and March 2025 at the Outpatient Diabetes Care Facility of the Pius Brinzeu County Emergency Hospital in Timișoara, Western Romania. This study focused on adult patients diagnosed with diabetes mellitus (both type 1 and type 2), aiming to explore behavioral and systemic determinants influencing dental care access using Andersen’s Behavioral Model of Health Services Use. The study protocol was approved by the Ethics Committee of the Victor Babeș University of Medicine and Pharmacy, Timișoara (approval no. 05/30.01.2024), and adhered to the principles of the Declaration of Helsinki. This study included adult patients with either type 1 or type 2 diabetes mellitus. This approach reflects the patient population commonly encountered in routine outpatient care, where both subtypes require continuous management and are subject to similar systemic, behavioral, and financial challenges affecting oral health. Given the shared complications and access barriers across both diabetes types, their combined analysis provided a more comprehensive evaluation of oral health determinants within the diabetic population.

### 2.2. Sample Size

A total of 160 patients attending scheduled consultations were approached, of whom 145 consented to participate. After applying eligibility criteria and excluding incomplete records, the final sample consisted of 79 participants with confirmed diagnoses of either type 1 or type 2 diabetes mellitus and complete clinical and questionnaire data.

For the present analysis, a subsample of 79 individuals with complete questionnaire (Supplementary S1) and clinical data was used. The sample size was estimated using G*Power 3.1 software, applying a point-biserial correlation model with an assumed 50% prevalence of oral health issues, a 95% confidence level, and a 5% margin of error. An additional 20% was added to account for potential non-responses. Stratified random sampling ensured representation across age groups, gender, and residential areas (urban vs. rural), aligning with the diabetes prevalence rate of 8.23% reported in the PREDATORR study [28,29].

### 2.3. Inclusion/Exclusion Criteria

Inclusion criteria were adult patients aged 18 years or older with a confirmed diagnosis of diabetes mellitus (either type 1 or type 2) attending scheduled consultations at the diabetes outpatient clinic. Only patients who were able to understand the study objectives and provide informed consent were eligible. Participants also needed to have complete clinical and questionnaire data to be included in the final analysis.

Exclusion criteria were patients with uncertain or unconfirmed diabetes diagnoses; individuals with cognitive impairments or psychiatric conditions that could affect the ability to give informed consent or reliably complete the questionnaire; patients recently hospitalized for acute trauma, major surgery, or systemic infections; institutionalized individuals (e.g., in long-term care facilities); and pregnant or breastfeeding women.

Of the 145 patients who initially provided informed consent, 66 were excluded from the final analysis. Specifically, 31 participants had incomplete or inconsistently filled questionnaires, 19 lacked recent or complete periodontal clinical records, 11 had unclear or unconfirmed diabetes status, and 5 met exclusion criteria such as recent hospitalization, cognitive impairment, or institutionalization. These steps ensured that the final sample of 79 participants met all eligibility requirements and provided complete and reliable data for analysis. To minimize bias and preserve data quality, only participants with fully completed questionnaires and periodontal clinical records were included. A complete-case analysis approach was applied, and no imputation was performed. The applied criteria were selected to ensure a clinically stable and non-institutionalized adult population capable of accurate self-reporting and standardized periodontal assessment, consistent with WHO-recommended procedures for observational studies. The presence of other systemic comorbidities (e.g., cardiovascular disease, hypertension, osteoarthritis) was not used as an exclusion criterion. This decision was made to preserve the ecological validity of the sample and to reflect the multimorbidity often encountered in real-life diabetic populations.

### 2.4. Data Collection

Data collection was conducted by a team of healthcare professionals including a diabetologist and two dentists. After providing informed consent, participants completed a self-administered questionnaire while awaiting their medical consultation. To minimize social desirability bias, the questionnaire was self-administered by participants in a designated area of the diabetes center, separate from clinical offices and without the presence of healthcare staff. Participants were clearly informed that their responses were confidential, anonymous, and would not influence their medical care. Additionally, the items were carefully worded using neutral non-directive language to encourage honest and accurate reporting.

The questionnaire was custom-developed for this study based on the WHO Oral Health Surveys: Basic Methods (5th edition) and informed by items previously validated in studies on diabetic populations in Romania [3,4]. It comprised 18 structured questions grouped into five thematic areas: demographic characteristics, oral hygiene behavior (e.g., brushing frequency, fluoride use), perceived oral symptoms (e.g., bleeding, sensitivity), barriers to accessing dental care (e.g., cost, fear, distance), and awareness regarding the bidirectional relationship between diabetes and oral health. The structure was designed to align with Andersen’s Behavioral Model, categorizing responses into predisposing, enabling, and need-related factors. Prior to full-scale implementation, the questionnaire was pilot-tested on a small group of diabetic patients (n = 10) to evaluate clarity, comprehension, and relevance. Feedback from the pilot phase led to minor wording adjustments. The core structure of the instrument was based on validated items previously used in Romanian diabetic populations [3,4]. While no formal psychometric validation was conducted for this study, the questionnaire was reviewed by a panel of academic researchers and clinicians to ensure content validity and cultural appropriateness.

The OHIP-14 instrument was chosen due to its concise format, international applicability, and established psychometric properties. The Romanian version, validated by Slusanschi et al. (2013), was culturally adapted and demonstrated high internal consistency (Cronbach’s α > 0.80) in adult populations. Its prior use in studies involving Romanian patients with diabetes further supports its contextual relevance and methodological suitability for evaluating OHRQoL in this population [30]. Items were scored on a 5-point Likert scale (0 = never to 4 = very often), with total scores ranging from 0 to 56 and higher values indicating greater impairment.

Participants were also evaluated clinically, with data collected on probing depth, plaque index, bleeding on probing, and attachment loss. Clinical examinations were performed by two calibrated dentists (I.A. and V.B) following WHO standardized periodontal assessment criteria. Prior to data collection, the two dentists conducting periodontal assessments participated in a calibration session involving five non-study patients. Inter-examiner reliability was evaluated using intra-class correlation coefficients (ICCs) for probing depth and attachment loss, yielding values above 0.85, which indicates a high level of agreement. This calibration procedure ensured standardized clinical measurements across the study. All clinical data were recorded on standardized paper forms and subsequently entered into electronic databases by two independent researchers. Cross-checking procedures were implemented to detect and resolve potential transcription errors, ensuring the accuracy of the dataset. A UNC-15 periodontal probe was used to assess probing depth, clinical attachment loss, plaque index, and bleeding on probing. Glycated hemoglobin (HbA1c) values were obtained from recent laboratory records to assess glycemic control and its association with periodontal parameters. A senior diabetologist from the research team (S.P.), who actively practices at the outpatient facility, contributed to participant selection and supervised the verification of clinical data, including HbA1c records and diabetes diagnosis. This ensured both clinical relevance and data accuracy throughout the recruitment process.

Income and insurance-related data were inferred from self-reported monthly earnings and employment status, as well as stated reasons for avoiding dental visits (e.g., cost, lack of reimbursement). While direct measures were not collected, these proxy indicators were aligned with official national income brackets and public health insurance eligibility thresholds. Given cultural sensitivities and privacy concerns frequently reported among older Romanian adults when disclosing financial details [31,32], income was inferred from employment status, education level, and reported financial barriers to care. This indirect approach aimed to minimize non-response bias while preserving socioeconomic relevance.

In line with Andersen’s Behavioral Model of Health Services Use [33], all variables were categorized into (1) predisposing factors (age, sex, awareness), (2) enabling factors (residence, employment status, inferred income), and (3) need-related factors (clinical oral symptoms, periodontal indicators, HbA1c levels).

### 2.5. Statistics

Data analysis was performed using SPSS version 23 (IBM, Armonk, NY, CA, USA). Descriptive statistics were calculated for all variables. Bivariate relationships between awareness, behaviors, and outcomes were assessed using Kendall’s tau and chi-square tests. Binary logistic regression models were used to identify predictors of dental service utilization and oral health symptoms, with odds ratios (ORs) and 95% confidence intervals (CIs) reported. Multivariable adjustment was applied in these models. Predictor variables were selected a priori based on their theoretical relevance to Andersen’s Behavioral Model and empirical support from the previous literature. The final models included awareness level, monthly income, place of residence, and HbA1c to assess their independent association with gingival bleeding. Pearson’s correlation coefficients were used to explore associations between continuous variables, including OHIP-14 scores and clinical or behavioral indicators. Statistical significance was defined as *p* < 0.05.

## 3. Results

### 3.1. Participant Characteristics

The study sample consisted of 79 adult individuals (ranging from 28 to 83 years) with a confirmed diagnosis of diabetes of either type 1 or type 2 diabetes mellitus attending scheduled consultations in a public diabetes outpatient clinic. The cohort had a mean age of 61.2 years (SD = 10.98), indicating an older population, with a significant proportion of participants aged 60 years and above (Table 1). This age profile reflects the chronic and progressive nature of diabetes, as well as the demographic trend toward population aging.

Females represented 55.7% of the sample, while males accounted for 44.3%. Regarding diabetes type, 86.1% (n = 68) of participants were diagnosed with type 2 diabetes mellitus (T2DM), while 13.9% (n = 11) had type 1 diabetes mellitus (T1DM). The average glycated hemoglobin (HbA1c) level across the sample was 8.24% (SD = 1.62), indicating that many patients had suboptimal glycemic control at the time of data collection. These characteristics are consistent with the epidemiological burden observed in similar outpatient populations and form the foundation for evaluating oral–systemic health interrelations.

Of the 79 participants, only 11 (13.9%) had a diagnosis of type 1 diabetes, while the vast majority (86.1%) were diagnosed with type 2 diabetes. Given this imbalance, no formal statistical comparisons between diabetes types were performed. However, descriptive patterns showed that patients with type 1 diabetes tended to be younger and had slightly better glycemic control, while oral health outcomes appeared broadly similar across diabetes types.

### 3.2. Predisposing and Enabling Factors

In line with Andersen’s Behavioral Model, participants’ predispositions and resource-based enabling factors were analyzed to better understand the contextual barriers to oral healthcare access. Predisposing elements included demographics, diabetes type, and awareness of oral complications. Awareness regarding the bidirectional relationship between diabetes and oral health was found to be insufficient in the majority of cases, with only 35.4% of respondents indicating a satisfactory understanding. This suggests a significant gap in education and communication from healthcare providers regarding oral–systemic links.

Enabling factors were assessed based on residence, occupation status, and inferred income levels. The urban-to-rural ratio was relatively balanced, with 58.2% of participants living in urban areas and 41.8% in rural settings, reflecting the sampling strategy aimed at capturing healthcare disparities across different residential environments.

Over 50% of participants were retired, which may limit their ability to seek routine dental care due to fixed or low income. Monthly income was categorized into four brackets: 32.9% reported earnings below RON 1500, 39.2% earned between RON 1500 and 2999, 17.7% fell within the RON 3000–4999 range, and only 10.1% exceeded RON 5000. These financial patterns align with national data on minimum pension and low-income thresholds, underlining the economic vulnerability of this population and its potential impact on health-seeking behaviors and treatment affordability (Table 2).

Education level was also recorded as a key predisposing factor. Among the study participants, 45.6% reported having completed secondary education, 36.7% had tertiary or university-level education, and 17.7% had only primary education.

### 3.3. Clinical-Need-Related Indicators

The analysis of clinical-need-related indicators, as defined within Andersen’s framework, included both subjective symptoms and objective clinical assessments. Self-reported oral symptoms were prevalent: 49.4% of participants experienced gingival bleeding during toothbrushing, 38.0% reported dental sensitivity, and 25.3% noted noticeable tooth mobility. These indicators reflect a high burden of oral inflammation and possible periodontal deterioration.

Clinical periodontal evaluation provided further evidence of this oral disease burden. The mean probing depth was 3.99 mm (SD = 0.86), while the mean clinical attachment loss was 4.65 mm (SD = 1.17), indicating a moderate level of periodontal tissue destruction in the study population. The plaque index averaged 31.28% (SD = 30.18), and bleeding on probing was observed in 37.73% (SD = 31.96) of sites examined. These values are suggestive of moderate-to-severe periodontal involvement in a significant portion of the study population, consistent with the chronic inflammatory response seen in diabetic patients with inadequate oral hygiene or limited access to preventive care.

Collectively, these results emphasize the existence of substantial unmet oral health needs and the presence of systemic, behavioral, and economic factors that may hinder access to care. The integration of these findings within Andersen’s model highlights the multidimensional complexity of health disparities in vulnerable patient populations (Table 3). When asked about their dental care history, only 27 participants (34.2%) reported having visited a dentist for treatment purposes within the past 12 months. A further 22 individuals (27.8%) reported attending only in cases of emergency (e.g., pain, swelling), while the remaining 30 participants (38%) had not sought any dental care in the past year.

### 3.4. Regression Analysis of Predictors

Binary logistic regression was conducted to identify the main factors associated with the occurrence of gingival bleeding, a frequently reported symptom reflecting periodontal inflammation. The analysis included four predictors: glycated hemoglobin (HbA1c), income level, place of residence (urban vs. rural), and self-reported awareness of the relationship between diabetes and oral health.

**Figure 1:**
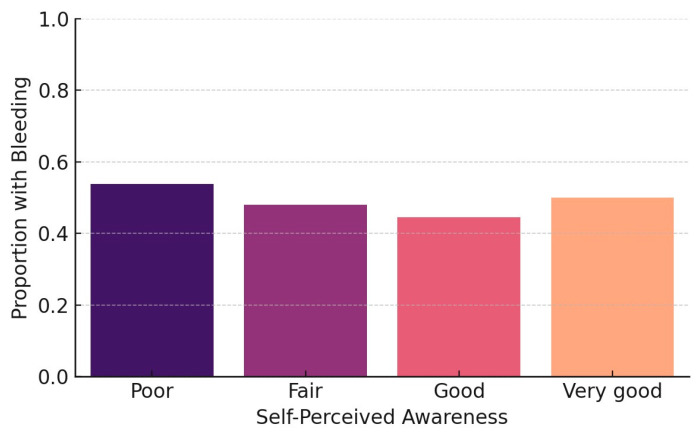
Prevalence of gingival bleeding by awareness level.

The final model revealed two statistically significant associations. Participants with limited or no awareness regarding the oral complications of diabetes were significantly more likely to report gingival bleeding (OR = 2.21, *p* = 0.033), highlighting the role of health education as a key behavioral determinant in periodontal symptom expression (Figure 1).

Second, low monthly income also emerged as a significant predictor. Individuals in the lower income brackets showed a higher probability of experiencing gingival bleeding compared to those with higher income levels (OR = 1.89, *p* = 0.041). This suggests that economic vulnerability may indirectly impact oral health by limiting access to preventive care or oral hygiene resources.

**Figure 2:**
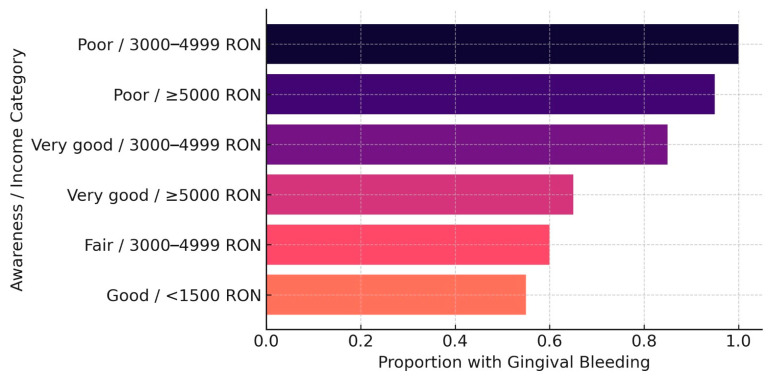
Top predictive groups for gingival bleeding.

A chi-square test was used to examine the association between oral health awareness and gingival bleeding. The result confirmed a statistically significant relationship (χ^2^ = 6.14, *p* = 0.046), further supporting the influence of health knowledge on oral symptom recognition. These findings align with previous evidence suggesting that awareness functions as a behavioral determinant in oral-health-related quality of life (Figure 2).

In contrast, HbA1c levels and rural residence did not show statistically significant associations with the outcome variable in this sample. Although these factors may contribute to general health disparities, their isolated predictive value for self-reported oral symptoms was not supported by the present data.

Overall, the regression results support the conceptual relevance of Andersen’s Behavioral Model. Both predisposing factors (awareness) and enabling factors (income) were shown to directly influence clinical need expression in diabetic patients, particularly with regard to early indicators of periodontal disease.

### 3.5. Oral-Health-Related Quality of Life Assessment (OHIP-14)

The impact of oral health on patients’ quality of life was evaluated using the Romanian version of the OHIP-14 instrument. The total OHIP-14 scores ranged from 0 to 48, with a mean value of 8.08 (SD = 11.32) and a median of 3. Over 50% of the participants reported very low OHIP-14 scores (≤3), indicating minimal perceived oral health impairment. However, a substantial minority scored above the 75th percentile (≥12), suggesting a moderate-to-severe impact on daily functioning, emotional well-being, or social interaction due to oral health issues.

**Figure 3:**
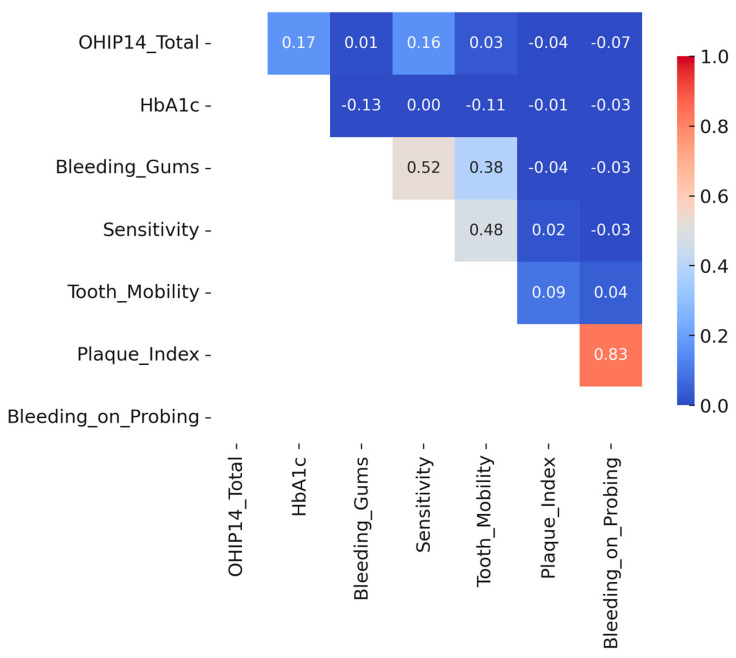
Correlations between OHIP-14 and oral health variables in diabetic patients.

To further explore the relationships between oral-health-related quality of life and clinical as well as behavioral factors, a correlation matrix was constructed using Pearson’s coefficients (Figure 3). The analysis revealed weak positive correlations between OHIP-14 total scores and both glycated hemoglobin (HbA1c) levels (r = 0.17) and self-reported dental sensitivity (r = 0.16), suggesting a modest association between poor glycemic control or oral discomfort and reduced perceived oral health quality of life. A weak inverse correlation was also observed between education level and OHIP-14 total score (r = –0.19), suggesting that individuals with lower education levels tended to report slightly poorer oral-health-related quality of life. Similarly, participants with lower education levels showed a marginally higher plaque index and bleeding on probing scores, although these differences were not statistically significant. These trends suggest that educational attainment may play a role in shaping both subjective and clinical oral health outcomes. In contrast, no significant associations were found between OHIP-14 scores and objective periodontal indicators such as plaque index, bleeding on probing, or probing depth. These findings reinforce the notion that perceived oral symptoms, rather than clinical markers alone, may more strongly influence patient-reported quality of life outcomes in diabetic individuals.

When examining associations with oral health outcomes, a weak inverse correlation was observed between education level and OHIP-14 total score (r = –0.19), suggesting that individuals with lower education levels tended to report slightly poorer oral-health-related quality of life. A similar trend was noted for clinical indicators: participants with lower education levels had marginally higher plaque indices and bleeding scores, although these correlations did not reach statistical significance.

## 4. Discussion

The complex interplay between oral health and systemic diseases—particularly diabetes—reflects shared risk factors, common inflammatory pathways, and overlapping socioeconomic determinants. Understanding these connections is essential for designing integrated care strategies. Drawing on recent research and global public health priorities, including the WHO 2030 vision, our findings highlight the urgent need for interdisciplinary collaboration, prevention-focused interventions, and systemic policy reform. Such efforts are crucial in reducing oral health disparities and improving overall health outcomes among vulnerable populations, particularly those living with chronic conditions like diabetes.

The oral cavity represents a critical interface between dentistry and general medicine, offering valuable insights into systemic health. Many systemic diseases—as well as the medications used to treat them—manifest oral signs or influence oral conditions.

Conversely, chronic oral diseases such as periodontitis may have far-reaching effects on systemic health. Associations have been observed between periodontal disease and conditions such as cardiovascular disease, diabetes, respiratory infections, kidney disease, osteoporosis, and adverse pregnancy outcomes. While the causal direction of these relationships remains under investigation, the bidirectional nature of the link is increasingly supported by clinical and epidemiological evidence. Both periodontal and systemic conditions share common characteristics: chronicity, inflammatory pathways, and long latency periods before symptoms become clinically apparent. The absence of early intervention often leads to significant complications, morbidity, and elevated healthcare costs. Unfortunately, limited access to medical and dental care—especially among vulnerable populations—delays diagnosis and management until critical events occur. These findings underscore the need for enhanced collaboration between dental and medical professionals to support early detection, integrated care, and prevention-driven approaches [34].

The bidirectional connection between oral and systemic diseases—particularly diabetes—has been increasingly acknowledged as a “two-way street,” where periodontal inflammation and glycemic imbalance mutually exacerbate one another [35]. This reinforces the need for multidisciplinary models of care that integrate dental and medical services, especially for vulnerable populations with chronic conditions. The present study aligns with this perspective by examining both clinical indicators and behavioral determinants within a health services framework.

Access to healthcare is shaped by a variety of structural and contextual factors, including the type of healthcare system in place, population-level education and health literacy, cultural attitudes, and income level [36]. Each country’s health system—organized under different financing and delivery models—acts as a key determinant of population health. In Nordic countries, for example, the emphasis on prevention results in substantial public investment in oral health promotion, routine check-ups, and early treatment programs [37,38]. By contrast, healthcare systems in Eastern Europe, including Romania, continue to face challenges related to underfunding and fragmented coverage.

Although our study did not directly assess health system coverage, previous national reports suggest that dental care accessibility in Romania remains limited due to structural and funding-related constraints. These broader system-level factors may contribute to the barriers observed in our diabetic sample [39]. These funding constraints directly affect access to dental services, which often fall outside the scope of reimbursed care. The situation is particularly severe in rural areas, where a lack of infrastructure and primary healthcare facilities further compounds access barriers. Health insurance coverage in these regions is limited, despite legal exemptions for specific population groups such as children and individuals with disabilities. Alarmingly, the number of insured persons continues to decline annually [39], exacerbating existing disparities. In this context, medically vulnerable groups such as older adults with diabetes are disproportionately affected, as their healthcare needs—particularly related to oral health—remain largely unmet by the public system.

Another barrier to accessing dental care is the method of payment for services. Individuals with higher socioeconomic status often choose to pay out-of-pocket for both preventive check-ups and curative treatments provided in private clinics. In contrast, those with lower socioeconomic status typically seek dental care only when urgent needs arise, such as in cases of pain or acute infections, thereby limiting their access to routine and preventive services [40]. This pattern reflects a key element of Andersen’s Behavioral Model, wherein enabling factors such as financial resources and perceived affordability significantly influence the decision to seek and utilize healthcare services, even when clinical need is evident.

Previous research has consistently demonstrated a strong association between low-income levels and adverse oral health outcomes, including an increased risk of oral cancer, a higher prevalence and experience of dental caries, tooth loss, traumatic dental injuries, periodontal disease, and diminished oral-health-related quality of life [41,42]. In our study, most participants reported modest financial resources.

Access to dental care in Romania is strongly influenced by structural factors such as insurance coverage, income level, and geographic location. Given that nearly 99% of dental services nationwide are delivered through private practices, financial barriers remain a critical issue, particularly for patients with lower socioeconomic status. Although our study did not explicitly assess whether participants sought care in private or public settings, the broader healthcare context suggests that individuals with modest or fixed incomes—such as retirees—are more likely to postpone or avoid routine dental visits due to anticipated costs. This interpretation is supported by our findings, in which fewer than 40% of participants reported attending dental appointments for treatment purposes, and a substantial proportion indicated financial constraints as a major barrier. These findings are in line with previous studies highlighting the influence of socioeconomic status on dental service utilization patterns [24,43]. These findings also align with the behavioral dimension of Andersen’s model, highlighting that clinical symptoms alone are often insufficient to prompt care-seeking behavior. Instead, limited financial means, low perceived need, and systemic barriers contribute to underutilization. The fact that many patients avoid routine dental visits—even in the face of evident oral health issues—emphasizes the need for targeted education and supportive policy measures to promote timely preventive care.

Gender-specific differences were also evident in care-seeking behavior. Women in the study more frequently cited dental anxiety as a primary deterrent, while men tended to mention cost and lack of time. These findings mirror prior research and highlight the role of psychological barriers—such as fear of pain, embarrassment, or negative past experiences—as key determinants of healthcare avoidance [24,44]. Addressing such barriers requires more than structural reform; it calls for patient-centered approaches that incorporate counseling, desensitization strategies, and communication training for dental professionals.

Our findings support existing evidence that links socioeconomic status to oral health behaviors and outcomes [45]. Participants with lower monthly incomes demonstrated poorer oral hygiene practices, reflecting limited engagement in preventive care. Interestingly, despite these deficient behaviors, most individuals perceived their own oral health as satisfactory and did not feel the need to seek dental treatment. This disconnection between actual clinical need and perceived oral health status may act as a behavioral barrier to accessing dental services. These findings support existing evidence that links educational attainment to both oral health behaviors and outcomes. Although statistical significance was not reached in our sample, participants with lower education levels reported higher plaque and bleeding indices and slightly worse OHRQoL scores. These trends suggest that lower educational attainment may act as a proxy for limited health literacy, reduced preventive behaviors, and diminished access to care, important considerations for future targeted interventions. Such patterns are likely influenced by a relatively low level of oral health education and awareness, particularly among adults in Romania. When combined with financial limitations, this contributes to poorer oral health outcomes compared to populations in countries with more robust preventive systems and higher public investment in dental health [24].

Older adults in rural areas are often exposed to multiple layers of vulnerability when it comes to healthcare access. Beyond financial barriers, rural populations are disproportionately affected by behavioral risk factors, including higher rates of smoking, alcohol use, sedentary lifestyles, and suboptimal nutrition [46,47,48]. These patterns contribute to an increased prevalence of NCDs, such as obesity, diabetes, cardiovascular disease, and cancer: conditions often considered preventable yet highly burdensome in underserved communities. Although our study did not focus exclusively on elderly individuals, a substantial proportion of participants were over the age of 60, and nearly 42% resided in rural areas. These participants reported lower income levels and less frequent use of dental services, consistent with a recent global analysis involving data from 42 low- and middle-income countries, linking rural residence and financial strain to delayed care. Our findings, combined with the previous literature, suggest that older adults with diabetes may face greater challenges in accessing oral healthcare, especially in contexts where public coverage is limited or inconsistent.

International data also suggest that diabetic patients in rural settings are 15–30% less likely to meet care benchmarks, highlighting systemic gaps in prevention and chronic disease management [49]. Moreover, even when healthcare services are technically available, rural patients tend to engage less frequently with the system, resulting in delayed management and poorer health outcomes overall.

The results align with Andersen’s Behavioral Model of Health Services Use. Although the clinical need for periodontal care is evident across the sample, access barriers—especially those linked to rural residence and low income—appear to restrict effective access to oral healthcare. Moreover, limited awareness and education about diabetes-related oral complications, as captured in the questionnaire, reflect additional predisposing challenges. Together, these findings highlight the complex interaction between systemic, behavioral, and clinical factors in shaping oral health disparities among individuals with diabetes in Romania.

A notable finding in our study was the disconnect between self-reported oral health and objective clinical indicators. Although more than half of the participants rated their oral health as satisfactory, a large proportion exhibited signs of periodontal disease, including bleeding gums and increased probing depth. OHIP-14 scores also revealed that subjective symptoms such as dental sensitivity had a greater impact on perceived quality of life than clinical markers like plaque index or attachment loss. These results reinforce the importance of subjective experience in guiding healthcare behavior and further validate the inclusion of patient-reported outcomes in both research and routine care. The lack of a strong correlation between clinical indicators (such as plaque index or probing depth) and OHIP-14 scores may reflect the limited awareness of patients regarding the clinical significance of periodontal symptoms. This mismatch has been documented in previous research and underscores the importance of addressing oral health literacy in diabetic populations when interpreting subjective quality of life measures.

Although some of the regression findings yielded only modest effect sizes, their clinical significance should not be overlooked. In medically vulnerable populations such as diabetic patients, even small improvements in awareness or socioeconomic status may translate into meaningful changes in preventive behaviors and service utilization. The predictors included in the model—awareness level, income, HbA1c, and residence—were selected based on their conceptual relevance within Andersen’s Behavioral Model and supported by the previous literature. By adjusting for these factors simultaneously, the multivariable analysis allowed us to better isolate their independent associations with outcomes such as gingival bleeding. These results emphasize the importance of modifiable determinants—particularly health literacy and financial capacity—in guiding intervention priorities for diabetic care.

From a public health perspective, these findings underline the urgent need for integrated, interdisciplinary approaches to diabetic care, approaches that include oral health education, routine screening, and access to affordable preventive services. In particular, dental professionals should be more actively integrated into diabetes care pathways. Dentists and periodontists can contribute by performing routine periodontal assessments, recognizing signs of poor glycemic control through oral symptoms (e.g., gingival bleeding, delayed healing), and offering tailored oral hygiene counseling. They are also well positioned to reinforce patient education on the bidirectional link between diabetes and oral health and to encourage compliance with both dental and medical follow-ups. Regular periodontal evaluations should be considered part of chronic disease monitoring. These roles highlight the value of a collaborative model where diabetologists, general practitioners, and oral health professionals work together to reduce complications and improve long-term outcomes in diabetic care. Dental care must be restructured to reach vulnerable groups, especially elderly and rural patients who often fall outside the reach of private-sector-dominated service delivery systems. Public policy should focus on expanding National Health Insurance coverage to include more preventive periodontal services and incentivizing public dental practices in underserved regions. Simultaneously, medical professionals, including diabetologists and general practitioners, must be trained to recognize oral–systemic links and refer patients proactively.

This study has several limitations that should be acknowledged. First, although the study was limited to one outpatient facility in Western Romania, the sample reflects the typical demographic and clinical profiles of diabetic patients receiving public care in the region. Nevertheless, caution is warranted when extending the findings to broader or nationally diverse populations, as healthcare access and patient characteristics may vary across different settings.

Second, the reliance on self-reported data introduces the possibility of recall bias and social desirability effects. Third, due to the cross-sectional nature of the study, causality cannot be established between behavioral factors and oral health outcomes. Additionally, while Andersen’s Behavioral Model offered a robust analytical structure, some key socioeconomic indicators—such as income and insurance coverage—were not directly measured. Instead, they were approximated based on self-reported earnings, employment status, and declared financial barriers to care. Although efforts were made to align these categories with national thresholds, this indirect approach may have introduced some degree of misclassification. While the study was based on a relatively small sample, the use of Andersen’s Behavioral Model provided a structured and validated approach to interpreting complex associations between systemic, behavioral, and clinical factors. The model’s relevance has been demonstrated in several recent studies focused on oral health inequities and chronic disease management, reinforcing its utility even in smaller-scale observational research [20,21,33].

Another limitation of the present study is that, although general medical comorbidities and pharmacological treatments were noted in patient records, they were not systematically analyzed. Future research should consider including detailed assessments of polypharmacy and multimorbidity, which are highly prevalent in elderly diabetic patients and may influence both oral health status and quality of life. Finally, the study population was recruited from a diabetes outpatient clinic, possibly excluding individuals with lower health-seeking behavior or poor access to care, thus introducing a potential selection bias. Future studies should incorporate direct measures of income, health insurance coverage, and out-of-pocket dental spending using standardized economic assessment tools. This would allow for a more accurate modeling of how financial capacity and structural access influence both clinical outcomes and oral-health-related quality of life in diabetic populations. In addition, complementing quantitative data with qualitative insights—through interviews or focus groups—could help uncover the emotional, cultural, and psychological dimensions of care avoidance. Adopting a mixed-methods design would allow researchers to explore in greater depth how diabetic patients perceive and navigate oral health challenges, providing a more comprehensive understanding of service utilization behaviors within vulnerable populations.

These observations support the implication that the presence of clinical need alone is insufficient to ensure service utilization. To promote timely and effective dental care among diabetic patients, efforts should focus on improving enabling conditions such as financial accessibility, geographic coverage, and patient awareness. In populations with chronic diseases like diabetes, oral health must become an integrated component of systemic disease management, not an optional extension. While the findings contribute to a growing body of evidence on oral health disparities in diabetic populations, they should be interpreted in light of the study’s observational and cross-sectional design. As such, the results offer exploratory insights rather than definitive evidence for clinical or policy decisions. In addition, given the small size of the type 1 diabetes subgroup (n = 11), the results of this study should not be extrapolated to the general T1DM population and must be interpreted with caution. Future research including larger and more balanced samples is needed to explore potential differences in oral health outcomes between T1DM and T2DM patients. Further longitudinal and multi-site studies are needed to validate these associations and guide the development of targeted interventions at the population level. Future research should consider longitudinal designs to track changes in oral health outcomes and perceived quality of life over time in diabetic populations. Given the need for the ongoing surveillance of systemic complications, integrating routine oral health evaluations into chronic disease management frameworks represents a natural and necessary extension of holistic patient care.

## 5. Conclusions

This study identified key behavioral, socioeconomic, and clinical factors influencing oral health outcomes and quality of life among diabetic patients in Romania. Regression analysis revealed that limited awareness and low income were significant predictors of gingival bleeding, while correlation findings showed that dental sensitivity and poor glycemic control had modest associations with impaired OHRQoL. Despite evident clinical need, many participants—particularly those from rural areas or with lower education levels—reported limited dental service utilization. These results suggest that future diabetes care models should integrate oral health components by addressing disparities through improved education, expanded access, and interdisciplinary collaboration. We recommend that oral health be formally integrated into chronic disease management frameworks, especially for older and socioeconomically vulnerable patients. In addition, longitudinal research is warranted to capture how oral-health-related quality of life evolves over time and to better assess the dynamic impact of behavioral interventions, glycemic control, and treatment adherence. Future research should build on these findings by adopting longitudinal designs, directly assessing socioeconomic status, and incorporating oral health education to better align clinical indicators with patients’ perceived quality of life.

## Figures and Tables

**Table 1:** Sociodemographic and medical characteristics of the study participants (N = 79).

Variable	Category	N (%)	Mean (±SD)
Age	34–44	5 (6.4%)	39.2 (±3.42)
	45–54	14 (17.9%)	50.5 (±3.18)
	55–64	24 (30.8%)	59.38 (±2.27)
	65–74	29 (37.2%)	69.34 (±3.17)
	75–87	6 (7.7%)	78 (±3.9)
Gender	Female	44 (55.7%)	
	Male	35 (44.3%)	
Diabetes Type	Type 1	11 (13.9%)	
	Type 2	68 (86.1%)	
HbA1c			8.24 (±1.62)

**Table 2:** Distribution of predisposing and enabling factors according to Andersen’s Behavioral Model.

Variable	Category	N (%)
Awareness	Satisfactory	28 (35.4%)
	Limited	26 (32.9%)
	None	20 (25.3%)
	Unclear/Others	5 (6.3%)
Residence	Urban	46 (58.2%)
	Rural	33 (41.8%)
Employment	Retired	40 (50.6%)
	Employed	39 (49.4%)
Income	<1500 RON	32.90%
	1500–2999 RON	39.20%
	3000–4999 RON	17.70%
	≥5000 RON	10.10%

**Table 3:** Self-reported oral symptoms, clinical periodontal indicators, and patterns of dental service utilization among diabetic participants.

Variable		N (%)	Mean (±SD)
	Bleeding gums	39 (49.4%)	
	Dental sensitivity	30 (38%)	
	Tooth mobility	20 (25.3%)	
	Probing depth (mm)		3.99 (±0.86)
	Attachment loss (mm)		4.65 (±1.17)
Pattern of Dental Service Utilization	Treatment visits in past 12 months	27 (34.2%)	
	Emergency visits only	22 (27.8%)	
	No dental visit in the past year	30 (38.0%)	

Note: Probing depth and clinical attachment loss are reported as mean values in millimeters (mm) ± standard deviation (SD). Higher values indicate more severe periodontal tissue destruction. Bleeding on probing and plaque index are expressed as percentages of sites examined.

## Data Availability

The data presented in this study are available on request from the corresponding author.

## Supplementary Materials

The following supporting information can be downloaded at: https://www.mdpi.com/article/10.3390/dj13060247/s1.

## Abbreviations

The following abbreviations are used in this manuscript:

OHRQoL	Oral-Health-Related Quality of Life
DM	Diabetes Mellitus
PD	Periodontal Disease
ABM	Andersen’s Behavioral Model
NCDs	Non-Communicable Diseases
OHIP-14	Oral Health Impact Profile—14 items
HbA1c	Hemoglobin A1c
NHIH	National Health Insurance House
SPSS	Statistical Package for the Social Sciences
SD	Standard Deviation
OR	Odds Ratio
CI	Confidence Interval
WHO	World Health Organization
UNC-15	University of North Carolina—15 mm probe
PREDATORR	Prevalence of Diabetes Mellitus and Cardiovascular Risk in Romania
