# Supplementary material for: Determinants of Oral Health Outcomes and Quality of Life in Diabetic Patients from Western Romania: A Behavioral Model Approach
Source: Dent J (Basel). 2025 May 30;13(6):247. doi: 10.3390/dj13060247 (PMC12192129; doi:10.3390/dj13060247)
Supplement: Supplementary file 1 [file dentistry-13-00247-s001.zip › dentistry-3635135-supplementary.pdf]

Patient Questionnaire: Diabetes and Oral Health Status, Behaviors, and Awareness

1. Age: ______ years
2. Gender: ☐ Male ☐ Female
3. Place of residence: ☐ Urban ☐ Rural
4. What type of diabetes do you have? ☐ Type 1 ☐ Type 2 ☐ I don't know
5. How long have you been diagnosed with diabetes? ☐ <1 year ☐ 1–5 years ☐ >5 years
6. How many natural teeth do you currently have in your mouth?
   ☐ 0 teeth, ☐ 1–7 teeth, ☐ 8–14 teeth, ☐ 15–20 teeth, ☐ 21–25 teeth, ☐ 26–28 teeth
   ☐ 29–32 teeth
7. How often do you brush with fluoride toothpaste your teeth? ☐ Once/day ☐ Twice/day ☐ Occasionally ☐ Never
8. Do you use dental floss or interdental brushes? ☐ Regularly ☐ Occasionally ☐ Never
9. Do your gums bleed when brushing? ☐ Yes ☐ No ☐ Sometimes
10. Do you experience dental sensitivity to cold or sweet stimuli? ☐ Yes ☐ No ☐ Sometimes
11. Have you noticed any tooth mobility? ☐ Yes ☐ No
12. When was your last dental visit? ☐ <6 months ☐ 6–12 months ☐ >1 year ☐ Never
13. What were the reasons for not visiting the dentist more often? (multiple answers possible)
    ☐ Financial reasons ☐ Fear ☐ Distance ☐ Lack of time ☐ No perceived need ☐ Emergency visit only ☐ Other: __________
14. How would you rate your current level of knowledge about the connection between diabetes and oral health (e.g., gum inflammation, periodontal disease, delayed healing)? ☐ Satisfactory, ☐ Limited☐ None ☐ Unclear/Others
15. Has your doctor or dentist ever explained the connection between diabetes and oral health? ☐ Yes ☐ No
16. Monthly income:
    ☐ <1500 RON ☐ 1500–2999 RON ☐ 3000–4999 RON ☐ ≥5000 RON ☐ Prefer not to say
17. What is your current employment status?☐ Employed ☐ Retired ☐ Unemployed
18. **What is your highest level of completed education?** ☐ Primary education (elementary school only), ☐ Secondary education (high school or vocational school), ☐ Tertiary education (college or university degree)
